# An ‘incredible community’ or ‘disgusting’ and ‘weird’? Representations of breastmilk sharing in worldwide news media

**DOI:** 10.1111/mcn.13139

**Published:** 2021-01-06

**Authors:** Sally Dowling, Aimee Grant

**Affiliations:** ^1^ Department of Nursing and Midwifery University of the West of England, Bristol Bristol UK; ^2^ Independent Researcher Cardiff UK

**Keywords:** breastfeeding, breast milk, human milk, mass media, qualitative research, stigma

## Abstract

Breastmilk sharing via the internet has become more popular in recent years, with a resultant increase in media attention. It is actively discouraged by public health bodies in at least three countries. We undertook a qualitative analysis of worldwide English language news media (online newspaper articles and transcripts of television and radio pieces) focusing on peer‐to‐peer breastmilk sharing during a 24‐month period (2015–2016). One hundred eleven news articles were analysed semiotically for positive (*n* = 49) and negative (*n* = 90) depictions of breastmilk sharing and the actors involved. Three countries published the majority of the articles: United States (*n* = 42), United Kingdom (*n* = 24) and Australia (*n* = 20). Topics associated with using shared breastmilk included perceived insufficiency, having surgery or taking medication, or the prematurity of the baby. Reports of women who *gave* and *received* breastmilk were largely positive although sometimes confused with women who *sell* breastmilk, who were demonised. The breastmilk itself, however, was considered as potentially contaminated and possibly dangerous; calls for action (*n* = 33) focused on increasing regulation and safety. Peer‐to‐peer milk sharing and the commercial availability of human milk are activities that occur within social and cultural contexts, and, as such, the ways in which they are represented in the news media reflect the ways in which they are also represented more widely in society. Increased understanding of normal infant feeding practices is needed, alongside guidance on how to better support breastfeeding. News media outlets can facilitate this through reporting risk in line with evidence. Further research should be undertaken to understand the safety of breastmilk sharing and the experience of those who participate.

**Key messages**

- Media organisations in the United States, United Kingdom and Australia reported regularly on breastmilk sharing in 2015 and 2016.
- Reports of milk *sharing* were often combined or confused with milk *selling*.
- Mothers who *gave* and *received* shared milk were mostly portrayed positive.
- Contradictory messages relating to the safety of shared milk were regularly reported, although these primarily appeared to be associated with *selling* milk.
- The media undermines informal breastmilk sharing; guidance should be developed to ensure appropriate reporting on breastmilk sharing.

## 1 INTRODUCTION

### 1.1 Background and current context

Breastmilk is recognised worldwide as the optimum food for babies and infants, whilst also providing health benefits for mothers and economic/other advantages for society (Pokhrel et al., 2014; Rollins et al., 2016; Victora et al., 2016). If a mother is unable to feed her baby from her breast, the hierarchy of alternatives include breastmilk from a healthy wet nurse and breastmilk from a milk bank (WHO/UNICEF, 2003), with feeding with artificial breastmilk substitutes (‘formula’ milk) (Victora et al., 2016) the least acceptable. Historically and cross‐culturally, informal breastmilk‐sharing arrangements have been the norm (Cassidy, Dykes, & Mahon, 2019; Cassidy & El‐Tom, 2010; Thorley, 2015) at times co‐existing alongside more formal arrangements (‘wet‐nursing’), although these have often disadvantaged marginalised, particularly Black, women and their babies (Palmer, 2009; Swanson, 2014). Regulated human ‘milk banks’ now exist in many countries—primarily to provide breastmilk to premature and unwell babies or in situations where the mother is unable to feed her baby herself (Cassidy et al., 2019; Swanson, 2014). Milk banks are usually reserved for feeding babies in very specific circumstances, in part because of the role of breastmilk in preventing serious infections in neonates but also because of issues of supply (milk banks receiving enough donations) and support for continuation of breastfeeding (Cassidy et al., 2019).

In recent years there has been an increase in research, using different methods, into how and why women choose to use other mother's milk through peer‐to‐peer arrangements (see, e.g., Akre, Gribble, & Minchin, 2011; Gribble, 2013; O'Sullivan, Geraghty, & Rasmussen, 2018). These are also sometimes referred to as informal‐, casual‐ or private‐arrangement milk sharing or as ‘milky matches’ (Carter & Reyes‐Foster, 2016; Cassidy, 2012; Palmquist et al., 2019). These arrangements are facilitated by the internet via social media and organisations such as Human Milk 4 Human Babies, as well as by the increase in availability of breast pumps and a culture of pumping (Boyer, 2010, 2014; Hausman, 2014), resulting in some women having an over‐supply and a desire to use rather than waste their excess milk. Milk sharing has also been shown to rely on personal and community contacts (Palmquist et al., 2019). Breastmilk is also exchanged as a commodity in commercial arrangements involving both individuals and corporations (Perrin et al., 2018); occasionally, this is for reasons other than feeding babies (Steele, Foell, Martyn, & Freitag, 2015).

### 1.2 Why use another woman's breastmilk?

There are physical and social reasons why women may not be able to breastfeed their babies themselves, or for as long as they would wish. Milk bank donations are not usually accepted from mothers of—or given to—older babies or used in community settings, being primarily reserved for feeding babies in very specific circumstances (Cassidy et al., 2019). Women's motivations to informally share or receive breastmilk outside of formal milk banking arrangements include the following: prematurity or illness, perceived insufficiency (Palmquist & Doehler, 2014; Stuebe et al., 2014), excess milk (Perrin et al., 2016), return to work and a lack of social or personal support (Cassidy et al., 2019; Palmquist et al., 2019). Some women want to continue providing breastmilk whilst avoiding the use of breastmilk substitutes (Gribble, 2013). An increased understanding of the role that breastmilk plays in healthy infant development has also contributed (Palmquist et al., 2019).

### 1.3 Understanding the use/sharing of human milk

Formal and informal breastmilk‐sharing scenarios exist alongside a range of cultural understandings of the meanings of women's bodies and, specifically, maternal milk. These affect how women's actions are perceived and influence societal understanding and acceptance of their behaviour, including in relation to the exchange and use of human milk (Cassidy, Dowling, Dykes, & Mahon, 2018; Cassidy et al. 2019; Kent, Fannin, & Dowling, 2019). Breastmilk sharing is undermined by the stigmatisation of breastfeeding in the global North (Tomori, Palmquist & Dowling, 2016; Grant, Mannay, & Morzella, 2017; Bresnahan, Zhu, Zhuang, & Yan 2019), the sexualisation of breasts (Dowling, Naidoo, & Pontin, 2012; Grant, 2016; Haucka, Bradfielda, & Kuliukasb, 2020) and the dichotomy whereby breastmilk is both perceived as dirty/‘matter out of place’ and as ‘liquid gold’ (Douglas, 2002 [1966]; Dowling, 2019)—contributing to the ‘yuk’ factor which may be invoked when discussing the use of another mother's milk (Shaw, 2004). Women who use other mother's milk may feel inhibited in discussing it, both because of these perceptions and because of their feelings about not being able to breastfeed their baby as they would wish (Esquerra‐Zwiers et al., 2016; Shafer, Ashada, & Palmquist, 2018).

Breastmilk sharing has been the subject of relatively recent academic attention, mostly from North America and Australia (e.g., see Gribble, 2013; Palmquist & Doehler, 2014, 2016). Important recent work includes the comprehensive review by Palmquist et al. (2019) and the collection of papers in the Supplement published by this journal in 2018 (Cassidy, Dowling, Dykes, & Mahon, 2018). Assessing the prevalence of informal milk sharing is difficult; this phenomenon has been investigated in the United States, for example, in a small study with 138 participants (Casser‐Uhl & Liberatos, 2018) and a mixed‐methods study with 41 participants providing qualitative data and 456 survey respondents (O'Sullivan et al., 2018). The latter found that 12% of the sample had provided their breastmilk to another mother and 7% had received it. Comparable research has not occurred in the United Kingdom. There is some evidence that commercial arrangements are more unusual than informal ones and that mothers feel more comfortable with the latter (O'Sullivan et al., 2018).

Other research has focused on outlining potential or perceived risks (Keim et al., 2014; Keim et al., 2015). Assessing the risk of using another mother's milk is not straightforward; some studies focus on commercial exchanges, others on commerce‐free situations, but perhaps not accurately replicating what happens in peer‐to‐peer exchanges (Palmquist & Doehler, 2016; Perrin et al., 2018). Informal milk sharing is not regulated, although in some (very few) countries, including Canada, the United States and France, there is public health guidance explicitly advising against the practice (Dowling, 2019). There is some evidence that women make careful decisions in relation to informal exchanges and assess potential risks using a range of available information (Palmquist & Doehler, 2016).

### 1.4 Media reports on human milk exchange

The practice of informal milk exchange using the internet is controversial (Gribble, 2018) and has increasingly been the subject of media discussion (Cassidy et al., 2018); views (reflecting those of society) are often polarised. Alongside this, health professionals are questioning their role in advising in this area (Steele, Martyn, & Foell, 2015; Dowling, 2019). The main issues of concern relate to potential/perceived health risks (Keim et al., 2013, 2014, 2015; Perrin et al., 2018; Steele, Foell, et al., 2015) and the involvement of strangers and potential associated risks, including contamination (Gribble, 2018).

As academics working in the United Kingdom we were interested in some high profile examples of the reporting of milk exchange. These included the media reports following the publication of Steele, Martyn and Foell's paper in 2015 (which was primarily referring to commercial practices and contained phrases such as ‘this market is dangerous, putting infant health at risk’) and the case of Ronja Wiedenbeck in 2016. Suddenly taken ill and unable to breastfeed her 11‐month old son, she appealed via Facebook for women to act as ‘wet nurses’ and was contacted by over a thousand women. Headlines in the media included ‘Model lets five STRANGERS breastfeed her baby boy’ (MailOnline, 11 April 2016). We noted that the media reaction to this story was varied and included some very positive and supportive reporting.

Carter and Reyes‐Foster examined the issue in the U.S. media (Carter & Reyes‐Foster, 2015; Carter & Reyes‐Foster, 2016) following the publication of work that highlighted potential risks associated with the practice (Keim et al., 2013). They found ‘complex and contradictory images of human milk’ and milk sharing. There has been no work looking at this more recently, or more widely geographically. The motivation for, and focus of, this paper therefore was to examine recent representations of breastmilk sharing worldwide in news media.

## 2 METHODS

### 2.1 Research Design

Our research adopted a qualitative documentary analysis, examining documents—in this case media articles—through an interpretativist lens (Grant, 2019) in order to understand how milk sharing was described in the English language media during a 24‐month period from 1 January 2015 to 31 December 2016. This time frame was chosen to encompass the two events identified above; our initial searches were carried out in 2017, and we searched for two whole years for completeness. Analysis of media content is a common form of documentary analysis because of the media's role in *creating* representations of acceptability and deviance (Hall, 1997). Our analysis specifically considered the semiotic portrayal of milk sharing, that is, whether milk sharing was considered to be inherently ‘good’ or ‘bad’, alongside examining discourses related to women who accepted and provided donor milk.

The rationale for this analysis approach is situated in light of there being no standardised analysis techniques within documentary analysis (Grant, 2019). However, in light of our research question, and the media's often binary (good/bad) reporting of people and events, we chose to use a semiotic approach to our coding and reporting of results. This approach fits with the dichotomous ways in which breastfeeding is often portrayed both in the media (Grant, 2015) and in real life (Grant et al., 2017). For example, a ‘good’ linguistic sign would be reporting a baby as ‘happy’ or ‘healthy’ and their mother as ‘positively surprised’; by contrast, ‘bad’ linguistic signs include references to breastmilk being ‘out of place’ in public. The use of cultural signifiers of shame and stigma are also relevant in relation to ‘bad’ representations of breastfeeding, reporting it to be shameful, dirty and thus required to be hidden in order to be polite. In addition to this, discourse analysis was utilised to understand the portrayal of individuals because, as demonstrated above, a significant body of research highlights that breastmilk sharing and the individuals involved can be demonised by the public, and we theorised—following Hall (1997)—that the media may contribute to the creation of this view.

### 2.2 Data Collection

English language news was collected using the newspaper indexing database *Nexis* (LexisNexis) during April 2017. This database, accessed via a university subscription, is a full‐text database of over 20 000 full‐text sources which includes U.K. national and regional newspapers and trade press as well as many newspapers and magazines published worldwide. European language sources are included in the database; other worldwide sources are English language. Articles which used terms related to milk sharing were captured, using a combination of a broad range of search terms (see Table 1). Search terms were developed through reviewing relevant literature and existing media cases. We purposely chose terms that did not prioritise the use of milk banks, as we were interested in informal peer‐to‐peer milk sharing. Classifying details were collected for each of the articles including the newspaper from which it was taken and the country from which it originated. Each article had a unique identification number, which was generated by Nexis and is retained by us in the reporting of the findings. The entire text of each article, along with its identification number, was copied and imported to NVivo 11 (QSR International) as an individual data entry, to facilitate analysis. Alongside this, a database of each included article, its identification number, month of publication and country of origin was created.

**TABLE 1:** Data collection strategy

Item	
Search terms	Milk sharing
	Breastmilk sharing
	Milk donation
	Breastmilk donation
	Donor milk
	Donor breastmilk
	Eats on Feets
	Human Milk 4 Human Babies
	Human Milk For Human Babies
	Milky matches
	Private arrangement milk sharing
	Peer to peer milk sharing
	Peer milk sharing
	Community milk sharing
Where should the search term be?	Anywhere in the text
News source	All English language news
Concept of interest	Sharing (donating) breastmilk online and via social media
	Seeking breastmilk online and via social media
Date range	1 January 2015 to 31 December 2016

### 2.3 Eligibility

Articles were eligible for inclusion if they were

- Focused primarily on peer‐to‐peer breastmilk sharing *and*
- Focused on breastmilk sharing for the benefit of infants *and*
- English Language

Articles were assessed against the eligibility criteria by one author [SD] and classified as not relevant, relevant and possibly relevant. The latter category was discussed by both authors before being assigned as relevant or not.

### 2.4 Analysis

Data were subjected to a semiotic and discourse analysis. The analysis assessed discourses focused on depictions of the actors involved and the context of milk sharing events or opinions in relation to the baby, the mother and the milk donor. Alongside this, news articles and sections of text within news articles were coded as ‘good’ or ‘bad’ in relation to their portrayal of milk sharing, based on linguistic signs and cultural signifiers, particularly in relation to infant health, maternal health, shame, stigma and appropriate behaviour (Eco, 1976; Mick, Burroughs, Hetzel, & Brann, 2004). Finally, ‘calls to action’ (Van Dijk, 2001), that is, suggestions of how breastmilk sharing should be changed, were considered, as in previous research on infant feeding (Grant, 2016). Coding was facilitated by QSR NVivo 11 software and was undertaken by both authors, with each coding a proportion of the articles. Sub‐codes within the semiotic and discourse analysis were discussed through regular data analysis meetings, in lieu of formal double coding. Themes were discussed as they were identified and clarified in line with content from all data within the study.

### 2.5 Researcher Positionality

Both authors have published work in relation to breastfeeding and milk sharing and approached the analysis of the data from the perspective of already being informed and interested in these issues. Author 1 is a qualitative researcher with a health professional background. Her research has focussed on a range of issues relating to infant feeding and breastfeeding experiences, including examining social and cultural influences on these. She has explored the experience of breastfeeding long‐term in her work and in doing this has drawn on her own experience, reflecting on this in her writing (Dowling, 2009, 2011; Dowling & Pontin, 2017). Author 2 is a qualitative researcher with expertise in documentary analysis (Grant, 2019, 2020) and public health, particularly focusing on pregnancy and infant feeding among working‐class British women. She has previously worked for Public Health Wales NHS Trust, focusing on infant feeding. Author 2 does not have any live children nor direct experience of breastfeeding; she has previously reflected on how this informs her research (Grant, 2018). She is a wheelchair user who is also Autistic, white, cis, heterosexual and married.

The chair of the relevant University ethics committee confirmed that ethical approval was not required for this study, as it was drawing on data from newspaper articles that were in the public domain and did not contain sensitive information.

### 2.6 Ethical statement

The chair of the relevant University ethics committee confirmed that ethical approval was not required for this study, as it was drawing on data from newspaper articles that were in the public domain and did not contain sensitive information.

## 3 FINDINGS

We first report our findings in relation to a description of the data collected and included in the analysis. Second, we provide a semiotic analysis of the key ‘actors’ involved: the mothers donating and receiving breastmilk and the babies. Third, we consider the way that breastmilk sharing as an activity is portrayed, both positively and negatively. Finally, we consider calls for action. In our reporting, we balance providing depth and breadth by including direct quotations, and—where the data are sufficiently broad—a breakdown of sub‐issues discussed within that data and the number (*n* = …) of cases.

### 3.1 Description of data

**FIGURE 1:**
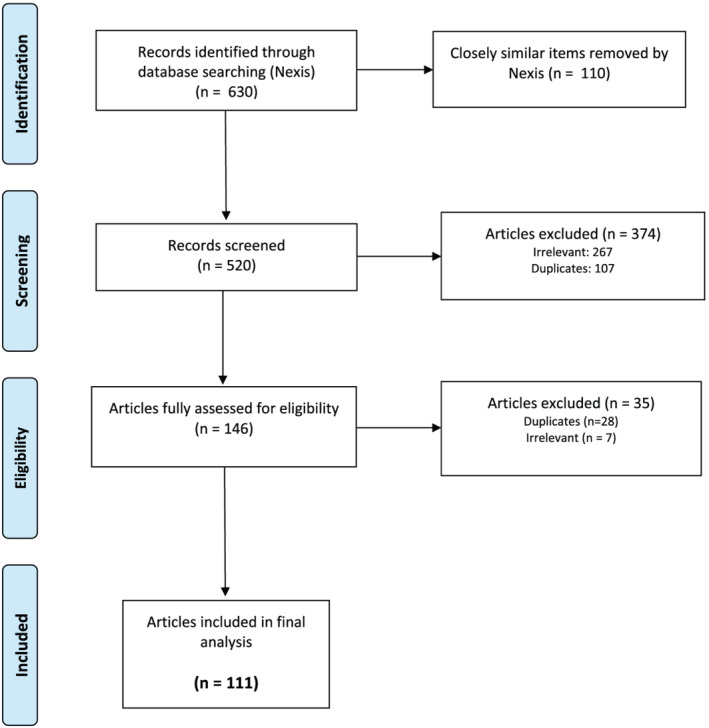
Process of identifying and selecting articles for inclusion in final analysis

Nexis identified 630 articles, removing those with close similarity (see Figure 1). As the same news story can sometimes be repeated—for example, in regional editions or to correct minor issues—with very little difference in text, Nexis looks for these duplications and removes them. Even after this process via the database we still found additional duplication, and the figure was reduced to 146 following further sifting for duplicates and irrelevant content. This was further reduced to 111 following discussion between the authors. The majority of irrelevant content focused on milk banking (*n* = 127) or infant formula being given to food banks (*n* = 36).

**FIGURE 2:**
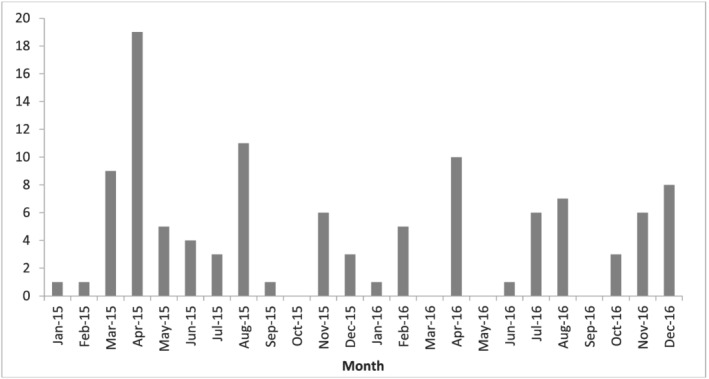
Number of English language articles on peer‐to‐peer breastmilk sharing published per month

The majority of the included articles originated from three countries (see Table 2): the United States (*n* = 42), United Kingdom (*n* = 24) and Australia (*n* = 20). Almost half (*n* = 49) were newspapers, with web publications and content from news agencies (‘news wires’) accounting for an additional 20 articles each. Clear peaks can be seen in the number of articles per month, with 3 months containing **≥**10 articles (see Figure 2).

**TABLE 2:** Description of included data

Descriptor	Sub‐code	*n*
News source	Newspaper	49
	Web publication	20
	Newswire (news agency)	20
	Newspaper and web publication	8
	Magazine	6
	Television transcript	6
	Blog	1
	Newsletter	1
Country	United States	42
	United Kingdom (of which Scotland = 4)	24
	Australia	20
	New Zealand	5
	Canada	4
	India	4
	Thailand	3
	France	2
	Ireland	2
	Singapore	2
	Armenia	1
	Cambodia	1
	China	1
Year	2015	64
	2016	47

### 3.2 Actors

Within our analysis, we considered *who* was the subject of attention. Mothers providing breastmilk, who we refer to as mother suppliers, and mother recipients featured heavily, both in their own right and the relationship between them.

#### 3.2.1 Mother suppliers

Mother suppliers were those women who offered their breastmilk to others. In the articles, they described themselves, or were described by health professionals who were quoted or the journalist writing the article, as having an excess supply of breastmilk (*n* = 22) and a desire to help mothers who struggled to breastfeed (*n* = 14) by *giving* their excess milk to mothers in need without payment. For example,

We are aware of the health benefits of breast milk; the form of baby feeding for centuries, and to donate milk to help other babies whom need it most is something I think many women will enjoy being a part of. ( 240)

Some women were directly motivated by their experiences of being unable to exclusively breastfeed, or the experiences of friends and family (*n* = 9). For example, one mother supplier wrote: ‘I've witnessed first‐hand friends battling with low supply, and the heartache and guilt they felt over the need to supplement.’ This mother supplier was described by the journalist as ‘the selfless mum’ (50).

This altruism was noted by health researchers and also by Netmums editor in chief:

Milk sharing is the ultimate milk of human kindness. In a world where almost everything is now commercialised it's wonderful to see families coming together to help and support each other for free. ( 351)

By contrast, some journalists and health officials also referred to mothers who *sold* their milk (*n* = 48) as creating a risky situation, akin to paid blood donors.

#### 3.2.2 Mother recipients

Mother recipients were those women who sought and received breastmilk from other women. Those who received shared milk were described in 63 articles; references were positive or neutral, identifying the mothers as blameless actors. A range of sympathetically worded reasons were given for mothers ‘needing’ to use shared milk. First, being ‘unable’ to breastfeed (*n* = 17) for vague reasons including ‘breastfeeding troubles’ and ‘new mums … having trouble nursing’ (332). More specifically, not producing ‘enough’ milk was explicitly referred to in 38 articles. The narrative of ‘my milk just dried up’ (5) expresses the sentiment in these articles, with no blame attributed to any actor or the maternal breast. Instead, correlation with premature birth and being unable to express sufficient milk was reported. In the example of one experienced breastfeeder, it was stated: ‘She tried hard to express milk but was only able to generate about 10 ml each day for her child’ who likely had a tongue tie (4). The sentiment of ‘Trying hard’ could be seen throughout these articles.

The second factor focused on being unwell, having breast surgery or taking medication (*n* = 23). Conditions were often not specified, such as using the term ‘pre‐existing medical conditions.’ (457). The more specific health conditions included were the following: being HIV positive, a blood clot on the brain; having had the body's anatomy changed through a mastectomy or gastric bypass, and cancer of the breast or colon. In one article the mental health of the mother was described, including the mother being autistic and having anxiety disorders. The medication contained in eight articles referred to chemotherapy or vague reports of medication that was ‘incompatible with breastfeeding’.

#### 3.2.3 Relationships between mother suppliers and recipients

The use of friends, wider peer groups and social media were described as introducing mother suppliers to mother recipients. Local parenting groups on Facebook facilitated milk sharing in four examples. Messages between mother recipients‐to‐be and potential suppliers were often heartfelt and full of kindness:

We were swamped. We had 50 people contacting us offering help. It was astonishing. We sat there in tears reading all these lovely messages. ( 11)

I'm so grateful and totally overwhelmed with the response to the message. It is such a loving and selfless act and incredibly heart warming to see. ( 185)

The Facebook group ‘Human Milk 4 Human Babies’ was specifically mentioned 39 times, another, *Eats on Feets*, 18 times. In addition to providing the milk, mother suppliers often went out of their way to help mother recipients, recognising that they were struggling (*n* = 14). For example, delivering breastmilk: ‘As (the mother recipient) lived a little further away, I'd just drop it when passing by. I like doing things that help people. It felt like a good thing to do.’ (173).

#### 3.2.4 Babies

Relatively little attention was paid to babies, with them featured in one third of articles (*n* = 40). The most common issue discussed was intolerance to infant formula or cow's milk (*n* = 13). For example, ‘“It started with a rash”, she recalls. “It was everywhere.” He soon began vomiting after feedings, and his weight plummeted.’ (429). Within 10 articles, prematurity was noted as a reason to require donor or shared milk on a temporary basis which would lead to the long‐term aim of exclusive breastfeeding once a mother's supply had been established: ‘It's a “bridge” that helps mothers supply an exclusive human milk diet’ (3).

### 3.3 Perceptions of milk sharing

#### 3.3.1 Milk sharing as inherently good

The majority of articles contained content that was both pro‐milk and anti‐milk sharing, with few representing only one point of view. Positive aspects of milk sharing included benefits for babies and the recognition of positive support networks between women.

Positive comments regarding milk sharing were made within 49 of the articles; 40 of these highlighted the general benefits of breastfeeding and/or breastmilk. It was not always specifically stated why breastmilk was considered better, content related to infant ‘health’ or ‘wellbeing’ or the ‘normal’ and ‘natural’ nature of breastfeeding. More specific positive factors included the following: helping to boost brain development, boosting immunity, reducing infection, increased recovery from illness and reducing maternal risk of breast cancer. The most positive of these extracts firmly positioned breastmilk as providing benefits to child health:

“Breast milk, because of its immunological properties, can help fight against infections that a baby may be exposed to,” said WebMD paediatrician (name). “It also may protect against allergies, asthma and sudden infant death syndrome.” ( 437)

Furthermore, 13 articles specifically highlighted the inferiority of infant formula compared to breastmilk, including

They don't develop their gut appropriately, which has implications for their immune system and lifelong health. ( 127)

Milk sharing was highlighted as often altruistic act between women, who provided mutual support to each other in 10 articles. The women involved were referred to as part of an ‘incredible community’ (5); one mother stated ‘They say it takes a village to raise a child, and that's certainly what's happening with our (baby)’ (11). The positive impact of this on mothers who were not able to feed their own children was often noted:

(the mother) was reduced to tears of joy as she was bombarded with nearly 1,000 offers from women all over the country offering to feed him … She was “totally overwhelmed” after receiving the kind responses. ( 185)

Other mothers who received donor milk were described as ‘grateful’, ‘very appreciative’ and ‘blessed’. In relation to risk, it was noted that mothers were vigilant and would not use breastmilk they considered potentially contaminated. Some mothers who received shared milk noted routine HIV screening during pregnancy and trusted donors to tell them if they had any infectious diseases. The particular benefits of donor milk for premature babies were described in four articles.

#### 3.3.2 Milk sharing as inherently bad

The most common negative depiction was milk sharing as an unsafe practice putting babies at risk (*n* = 84). Shared milk was reported to be more risky than milk obtained from a donor milk bank in half of these cases (*n* = 42). In 11 instances, this was explicitly linked to a lack of regulation of milk sharing, using statements like ‘Breast milk is also NOT regulated.’ (180). The major reported concern centred on the lack of screening for infectious diseases or bacterial contamination (*n* = 86) which would occur with milk received through a formal milk bank. Alongside this, the potential for lifestyle factors as a risk was highlighted (e.g., alcohol, smoking, drugs, personal hygiene) (*n* = 19). As a response to these perceived risks, a minority of articles (*n* = 6) suggested that infant formula was safer than milk sharing.

Furthermore, some articles contained both negative and positive viewpoints, with negative depictions often positioned alongside the positive descriptions of breastmilk sharing identified above. Within 90 of the articles, milk sharing was positioned as dirty, sexualised or risky. This is perhaps, in part, related to the practice of conflating both *selling* and *giving* under the banner of ‘milk sharing’. Milk selling, as a commercial practice, was highly stigmatised. Recipients faced feedback that milk sharing was ‘disgusting’ or ‘weird’, with social media platforms sites for receiving negative reactions. Women who sold their milk also experienced stigma and often attempted to hide their role, with authors describing them in negative terms:

(buying expressed breastmilk) is like buying a used toothbrush. For all we know, Mother's Little Helper may knock back a fifth of vodka a day or suffer from some loathsome disease. ( 444)

Stigma was most often reported by recipients to come from friends and family:

“There are still mixed emotions about it. Even among my friends, I have friends that think it's disgusting,” she said. “People want to keep it private because of the ridicule. We adults put other [animals'] milk in our bodies.” ( 127)

I've got friends who think it's gross and say, “Why don't you put your baby on formula?” ( 219)

In one article it was noted that some individuals not involved in milk sharing viewed it as an inherently sexualised practice:

“This looks like … a porn film,” one man wrote of the image (of a woman feeding her infant and her friends' infant simultaneously). ( 350)

Other articles (*n* = 8) noted that adults were the recipients of some donor milk either because of sexual fetishes or because of purported benefits to bodybuilders.

### 3.4 Calls to action

Within 33 of the articles, suggestions were made for how mothers (fathers were rarely mentioned) or donors should act that went beyond approving or disapproving of milk sharing. The most prominent suggestion was that milk sharing should be regulated (*n* = 23), either through stricter enforcement of existing laws or through the introduction of new laws.

Four articles focused on introducing screening procedures, to detect infection or contamination:

There is also a largely shared view that it's important for donor milk to be thoroughly screened for bacteria, drugs and adulteration by cow's milk. ( 377)

Other calls to action included providing guidance for women on how to milk share (more) safely (*n* = 8) or requests for additional support for women to be able to meet their own breastfeeding goals (*n* = 4). The majority of the calls for action (*n* = 29) were based on the notion that milk sharing was undesirable or problematic. The remaining four requested additional donors came forward to provide milk to the many women who had not been supported to breastfeed their child for as long as they desired and wished to provide expressed donor milk instead.

## 4 DISCUSSION

Our focus has been on understanding representations of milk sharing in English language news media. Peer‐to‐peer milk sharing and the commercial availability of human milk are activities that occur within social and cultural contexts, and, as such, the ways in which they are represented in the news media reflect the ways in which they are also represented more widely in society. In the news media articles discussed in this paper, women—fathers were largely notable by their absence—and their actions were portrayed in conflicting (and often dichotomous) ways. Women were both wonderful and dangerous; their milk both life‐giving ‘liquid gold’ and matter out of place (Douglas, 2002 [1966]). This echoes previous research which identified shared breastmilk as dichotomous: ‘pure gold’ versus ‘fools' gold’ (Carter & Reyes‐Foster, 2016). Risk and stigma were directed towards buying and selling breastmilk, but discourses related to commercial practices were incorrectly interwoven with peer‐to‐peer milksharing, conflating these two practices. An example of this was the report about the American Academy of Pediatrics ‘clear’ policy on the dangers associated with feeding babies unpasteurized milk, which talked about ‘sharing … amongst friends or relatives’, the ‘unregulated breast milk industry’ and breast milk being ‘bought, sold and traded’ in consecutive sentences in the same short piece (420). Stigmatising words used in the articles included ‘disgusting’ and ‘dirty’—but at the same time breastfeeding was the focus of public health campaigns (‘breast is best’); milk sharing takes place within this wider, and sometimes confusing, context.

In many of the representations discussed here women were separated from the actions of their bodies; the failure to produce sufficient milk for a baby was seen as bodily failure and not a failing of the woman herself. This is unusual—and very much welcome—in a patriarchal victim‐blaming culture (Taylor, 2020). However, it can also be viewed in relation to the concepts of trust and risk. It seems that women are—at least superficially—trusted when they are ‘unable’ to provide breastmilk. However, when they *do* provide breastmilk, whether from their own body (self‐citation) or via a donor (Shafer et al., 2018 **)** they are trusted much less. A layer of risk is applied to discourses; no matter what the ‘problem’ or ‘risk’, it is portrayed as the mother's fault. This is common in representations of breastfeeding; for the most part [Grant et al., 2019; Williams et al., 2019], it is not the breastfeeding itself that is the problem but the social context in which it is occurring and a patriarchal victim‐blaming culture.

Interestingly, when both mother recipients and babies were discussed, the barriers to breastfeeding highlighted by these data—as well as identified in other studies (e.g., see O'Sullivan, Geraghty, & Rasmussen, 2016)—are recognised as commonly occurring in breastfeeding support and in the literature about this. Commonly attributed reasons for breastfeeding difficulties include inaccurate maternal perception of either lactation insufficiency or infant lactose intolerance and mother and health practitioner perceptions of whether or not medication is compatible with breastfeeding. These may lead to the cessation of breastfeeding (Gatti, 2008; Casser‐Uhl & Liberatos, 2018). Accordingly, it is unhelpful that our research identified that the media is further contributing to this misunderstanding in one third of articles in our data set. The need for improving societal support, including cultural and social acceptance and understanding, for breastfeeding is well recognised (Rollins et al., 2016; Unicef, 2016) and may remove the need for many breastmilk‐sharing situations.

Our decision to explore media depictions of breastmilk sharing as inherently bad or inherently good reflected the way this dichotomy played out in the media articles we analysed. In addition, these aspects were also present in the portrayal of the actors in the milk‐sharing interactions. Much of the focus was on physical bodily aspects (insufficient milk, lactose intolerance, the health benefits of breastfeeding); it is perhaps not surprising that the relational aspects of breastfeeding for the mother–baby dyad, which are often central now to breastfeeding promotion and to infant feeding messages overall (e.g., see UNICEF, 2013, 2019), were underplayed. In contrast, the relationship between the supplier and the recipient did receive attention—with words like ‘altruism’, ‘trust’ and ‘help’ being emphasised. This reflects what is known about milk sharing from the literature (Gribble, 2018; Palmquist & Doehler, 2016) which suggests that these are often exchanges between women who meet in person and for whom interpersonal relationships are important—rather than ‘strangers’ (who make news headlines such as those that first sparked our interest in this work). Gribble (2018) notes that ‘peer‐to‐peer milk sharing is a modern form of cooperative mothering’; this idea perhaps underpinned many of the news stories with ‘good’ aspects that we examined.

There was also an element in this data of depicting maternal subjects and their actions towards babies as dangerous and in need of surveillance (Lupton, 2012); this was demonstrated in the portrayal of milk sharing as risky. We note, with interest, that risk was considered to be taken by maternal, not paternal figures (with babies the potential recipients of the consequences of the risk), despite generally more egalitarian parental relationships in the countries represented. Calls for action focused both on this danger and on regulation as the required response. Evidence highlights that the risks associated with breastmilk sharing are not clear and are often related to a number of factors. The reductive way in which they are discussed in the news media does not allow for the subtleties shown when women are asked how they assess and mitigate risks, for example, by asking lifestyle and health screening questions, or that the risks are not the same if milk is shared with known women versus bought from strangers (Palmquist & Doehler, 2016). The implicit—and sometimes explicit—assumption was that infant formula is less risky, although the risks of formula use (compared to breastfeeding) can be significant (Gribble & Hausman, 2012). Overall, calls for action were often based on the view that milk sharing is inherently problematic and risky and thus that mothers require surveillance. There were not any particular trends by country in terms of subject, depth and quality of reporting. These calls for surveillance have echoes of the way that the bodies and behaviour of pregnant women are policed in many cultures (McCallum & Holland, 2018; Grant et al., 2017). These assumptions have been operationalised in a minority of countries which have introduced public health warnings against obtaining breastmilk informally (either for money or for free), usually with a focus on ‘danger’ or ‘risk’ (Dowling, 2019). Health professionals have little guidance on how to advise and support women who chose to use other women's milk.

Limitations to our data set, and therefore to our interpretations, include the inclusion of only English language news media, and the high proportion of data focused on three countries. Nexis, the database we used, did not allow users to access images which accompanied articles. Images are often used by the media to stigmatise and sensationalise (Hall, 1997), alongside the textual signs that highlight positive and deviant acts. Consideration of images which accompanied articles alongside them may have illuminated a stronger preference towards representations of ‘good’ or ‘bad’ actors.

## 5 CONCLUSION

Research into peer‐to‐peer milk sharing is in its infancy. There is little known about the prevalence of this practice in many countries or women's motivations for either giving or receiving breastmilk, and the research on potential risks is conflicting. Further research is required to ascertain prevalence and evaluate risk; accordingly, developing guidance on milk sharing, as has occurred in a minority of countries, is premature at this time. Analysing representations of milk sharing in worldwide English language news media has contributed to our understanding by highlighting the sensationalist and unhelpful ways in which the media report on peer‐to‐peer breastmilk sharing. This practice is not unique to breastmilk sharing, and breastfeeding more generally is dichotomised and sexualised in print media (Grant, 2015). Accordingly, standards for reporting on infant feeding should be developed, as has occurred in relation to reporting on suicide (IPSO, 2020) to ensure that the media do not unwittingly undermine infant health. As is now widely acknowledged, increased societal and policy support is needed in order to normalise feeding infants with human milk (Rollins et al., 2016; Unicef, 2016).

## CONFLICTS OF INTEREST

Sally Dowling has previously received funding from the Economic and Social Research Council and from South Gloucestershire and Wiltshire Local Authorities. She is a co‐recipient of two internal research awards from UWE, Bristol (Vice Chancellor's Challenge Fund). Aimee Grant has received funding from the National Institute for Health Research, Economic and Social Research Council, the Wellcome Trust ISSF and the Welsh Crucible Small Grant Scheme. She has also undertaken paid consultancy for Public Health Wales NHS Trust, where she previously held the role of Senior Health Promotion Practitioner. She is affiliated with the Action on Smoking and Health (ASH) Wales Cymru research committee, where she previously held the role of Research and Policy Officer.

## CONTRIBUTIONS

SD undertook a literature review, and SD and AG conceived the research design. SD undertook the searches and extracted data. SD and AG screened the titles and full text of papers for inclusion/exclusion criteria and undertook the qualitative analysis. SD drafted the introduction and discussion; AG drafted the methodology and results. Both authors approved the final version of the paper.
